# First case report of a novel KIF13A-ALK fusion in a lung adenocarcinoma patient and response to alectinib with a 4-year follow-up

**DOI:** 10.3389/fgene.2023.1289346

**Published:** 2023-12-13

**Authors:** Zheng Mo, Cunliang Cai, Jingjing Yao, Jingquan Zhao, Mingqiang Zhang, Hao Liu, Xiangdong Mu

**Affiliations:** ^1^ Department of Oncology, Beijing Tsinghua Changgung Hospital, School of Clinical Medicine, Tsinghua University, Beijing, China; ^2^ Department of Respiratory and Critical Care Medicine, Beijing Tsinghua Changgung Hospital, School of Clinical Medicine, Tsinghua University, Beijing, China; ^3^ Department of Pathology, Beijing Tsinghua Changgung Hospital, School of Clinical Medicine, Tsinghua University, Beijing, China

**Keywords:** anaplastic lymphoma kinase (ALK fusion), case report, kinesin family member 13A (KIF13A), lung adenocarcinoma, alectinib

## Abstract

The prevalence of Anaplastic Lymphoma Kinase gene (*ALK*) fusion is about 5% among patients with lung adenocarcinoma, underscoring the importance of pinpointing distinct fusion variants for optimizing treatment approaches. This is the first reported case of a 74-year-old female with stage IV lung adenocarcinoma, featuring a novel Kinesin Family Member 13A (*KIF13A*)*-ALK* fusion, identified via next-generation sequencing (NGS) and confirmed with fluorescence *in situ* hybridization (FISH). Initially undergoing chemotherapy and then crizotinib, she achieved a partial response (PR) before progressing with multiple bone metastases. However, subsequent treatment with alectinib as a third-line option yielded positive results. A stable disease state persisted for an impressive 31 months of progression-free survival (PFS), accompanied by minimal toxicity symptoms. Up until now, a remarkable near 4-year span of overall survival (OS) has been consistently observed and monitored. This report of a *KIF13A-ALK* fusion case benefit significantly from alectinib with extensive follow-up. The case diversifies the array of *ALK* fusion partners and holds clinical relevance in refining therapeutic choices for *KIF13A-ALK* fusion-associated lung cancer.

## Introduction

The therapeutic landscape of lung adenocarcinoma has undergone a profound shift, catalyzed by the discovery of diverse gene fusions that drive tumorigenesis. Among these, Anaplastic Lymphoma Kinase gene (*ALK*) rearrangements have emerged as pivotal targets for precision medicine due to their central role in oncogenesis. These well-described driver mutations exist in approximately 5% of non-small-cell lung cancers (NSCLCs) (Devarakonda et al., 2015). Patients with *ALK* fusions can experience substantial benefits from *ALK* tyrosine-kinase inhibitors (TKIs) like alectinib. Notably, it is imperative to recognize that distinct *ALK* fusion partners generate heterogeneous responses to *ALK* TKIs (Chuang et al., 2021; Sun et al., 2021). As a result, identifying specific fusion variants assumes paramount importance in refining treatment strategies. Kinesin Family Member 13A (*KIF13A*), a constituent of the kinesin superfamily of motor proteins, has garnered attention for its active participation in intracellular transport and cargo trafficking within cells (Zhang et al., 2018). Within the scope of this study, we present a noteworthy discovery: the identification of an unreported fusion involving *KIF13A* and *ALK* in a patient with lung adenocarcinoma. This *KIF13A-ALK* fusion displays exceptional sensitivity to alectinib treatment, leading to prolonged survival and an enduring favorable clinical response. In addition to advancing our understanding of the phenomenon, this report underscores the promising therapeutic implications for patients carrying this fusion.

## Case report

**FIGURE 1:**
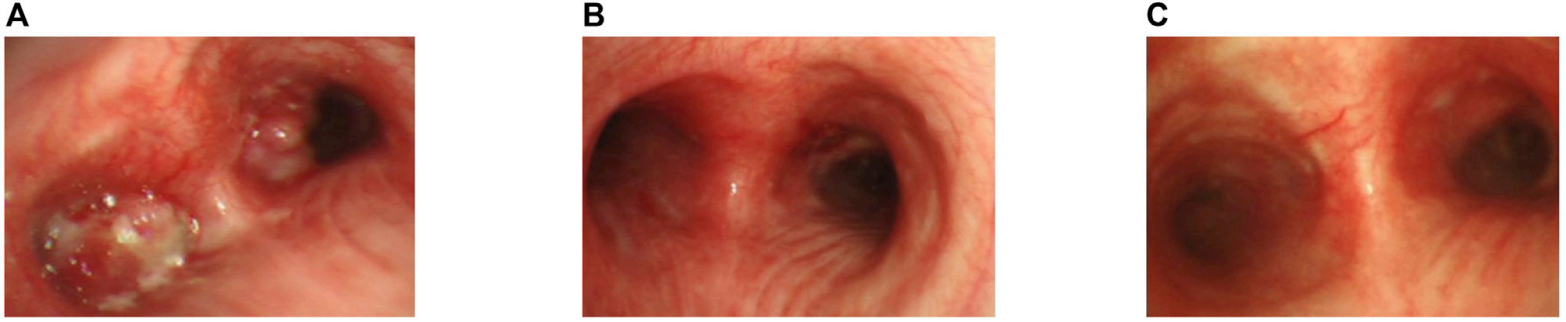
Bronchoscopic view of the patient. **(A)** Bronchoscopic view at the carinal level, illustrating obstructive masses within the bilateral bronchi and the left main bronchus.**(B)** After the apply of bronchoscopy electrocautery for central airway obstruction.**(C)** Two months following the commencement of alectinib treatment.

**FIGURE 2:**
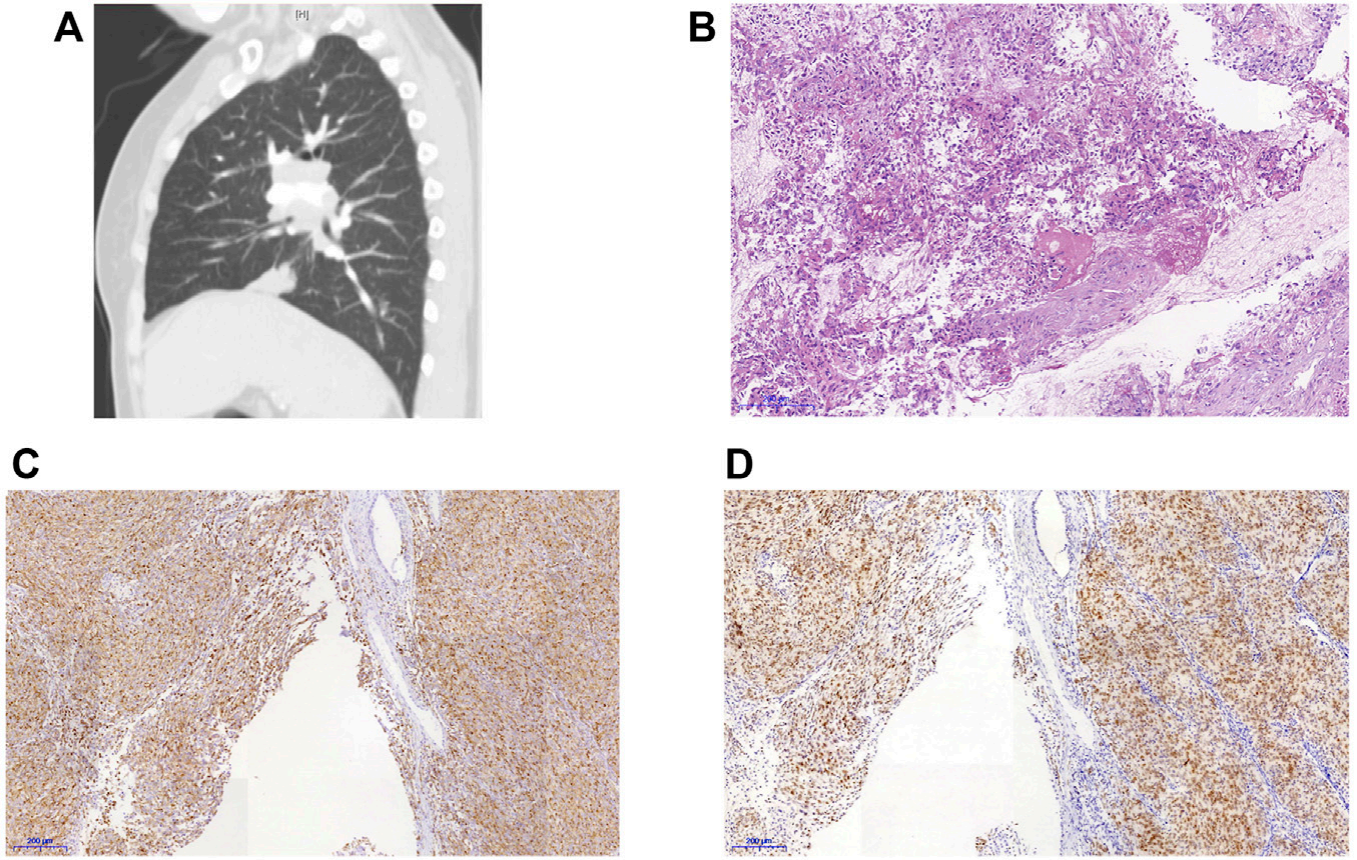
CT scan and lung biopsies of the reported patient. **(A)** CT findings of the patient **(B)**HE staining of the biopsy specimen, 40×. **(C)** Immunohistochemical staining for Napsin A. **(D)** Immunohistochemical staining for thyroid transcription factor-1 (TTF-1).

A 60-year-old non-smoking Chinese female was admitted to our hospital in October 2019 with a one-month history of cough, sputum production, and dyspnea. Computed tomography (CT) scan revealed pulmonary lesions in the left main bronchus and lower right lobe invading mediastinum, concomitant with widespread metastases involving multiple mediastinal lymph nodes, the liver, and multiple bones. Diagnostic bronchoscopy revealed an uneven and congestive polypoid mass with luminal obstruction of the right principal bronchus, electrocautery based on bronchoscopy was applied for central airway obstruction (Figures 1A, B). Chest CT and pathological examination of a bronchoscopic biopsy showed poorly differentiated invasive adenocarcinoma. (Figures 2A, B). Immunohistochemical (IHC) analysis revealed positive staining for Napsin A and thyroid transcription factor 1 (TTF1) which have been widely used as sensitive markers of pulmonary adenocarcinoma (2) (Figures 2C, D). The patient’s condition was classified as Stage IV (cT4N3M1c) adenocarcinoma.

**FIGURE 3:**
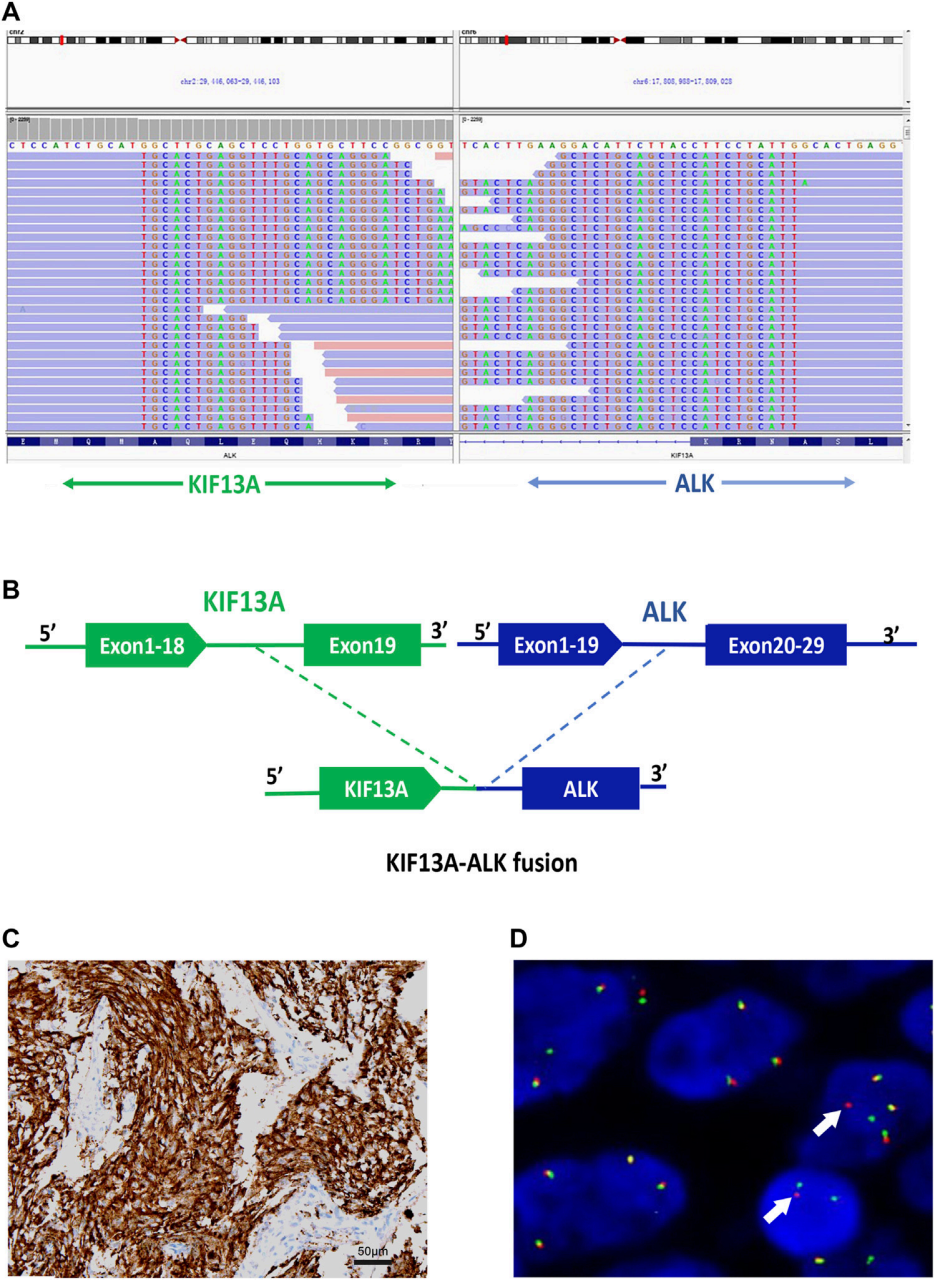
Identification of the KIF13A-ALK fusion **(A)** *KIF13A*-*ALK* fusion was validated manually using the Integrated Genomics Viewer; **(B)** A novel intergenic region between exons 1-18 of *KIF13A* on chromosome 6 and exons 20-29 of *ALK* on chromosome 2 fusion variant was identified. **(C)** Validation of *ALK* expression via IHC staining (40×), **(D)** Fluorescence *in situ* hybridization (FISH) analysis utilizing the *ALK* split apart probe, demonstrating distinct split signals (indicated by arrows) suggestive of a rearrangement of the ALK gene.

The tumor tissue acquired during biopsy was sent for genomic testing by next-generation sequencing (NGS) based on a pan-cancer 1021-gene panel containing whole exons and selected introns of 288 genes and selected regions of 733 genes, a novel *KIF13A-ALK* fusion was identified (abundance: 11.4%). This fusion encompassed exons 1-18 of *KIF13A* on chromosome 6 and exons 20-29 of *ALK* on chromosome 3 (Figures 3A, B). Notably, no concurrent driver alterations, including those in *EGFR, ROS1, BRAF, KRAS, MET,* or *RET* genes, were detected. Furthermore, immunocytochemistry (IHC) and fluorescence *in situ* hybridization (FISH) were also performed to verify the above mutation (Figures 3C, D).

Given the novelty of this *ALK* fusion, its specific response to *ALK* tyrosine kinase inhibitors (TKIs) remained uncertain, further accentuated by the patient’s lack of insurance coverage for *ALK* TKIs. After considering the patient’s preferences, a therapeutic approach comprising six cycles of first-line chemotherapy with pemetrexed (500 mg/m^2^ every 3 weeks) and cisplatin (75 mg/m^2^ every 3 weeks) was initiated in October 2019. Notably, complete resolution of symptoms was achieved. Subsequent evaluations via CT scan and bronchoscopy in December evidenced a partial response (PR) across all lesions, accompanied by nausea and a reduction in white blood cell count (2.2 × 10^9^/L) classified as grade 2 according to Common Terminology Criteria for Adverse Events (CTCAE) version 5.0. Regrettably, following the completion of six cycles of chemotherapy, the patient experienced disease progression (PD) attributed to the emergence of additional bone metastases. In response, the patient was transitioned to crizotinib at a dose of 250 mg twice daily. However, after 1 month of crizotinib treatment, imaging studies revealed further progression of bone metastases. Consequently, crizotinib was discontinued, and the patient was subsequently administered alectinib therapy (600 mg twice daily) in conjunction with radiation therapy for thoracic vertebral metastasis.

**FIGURE 4:**
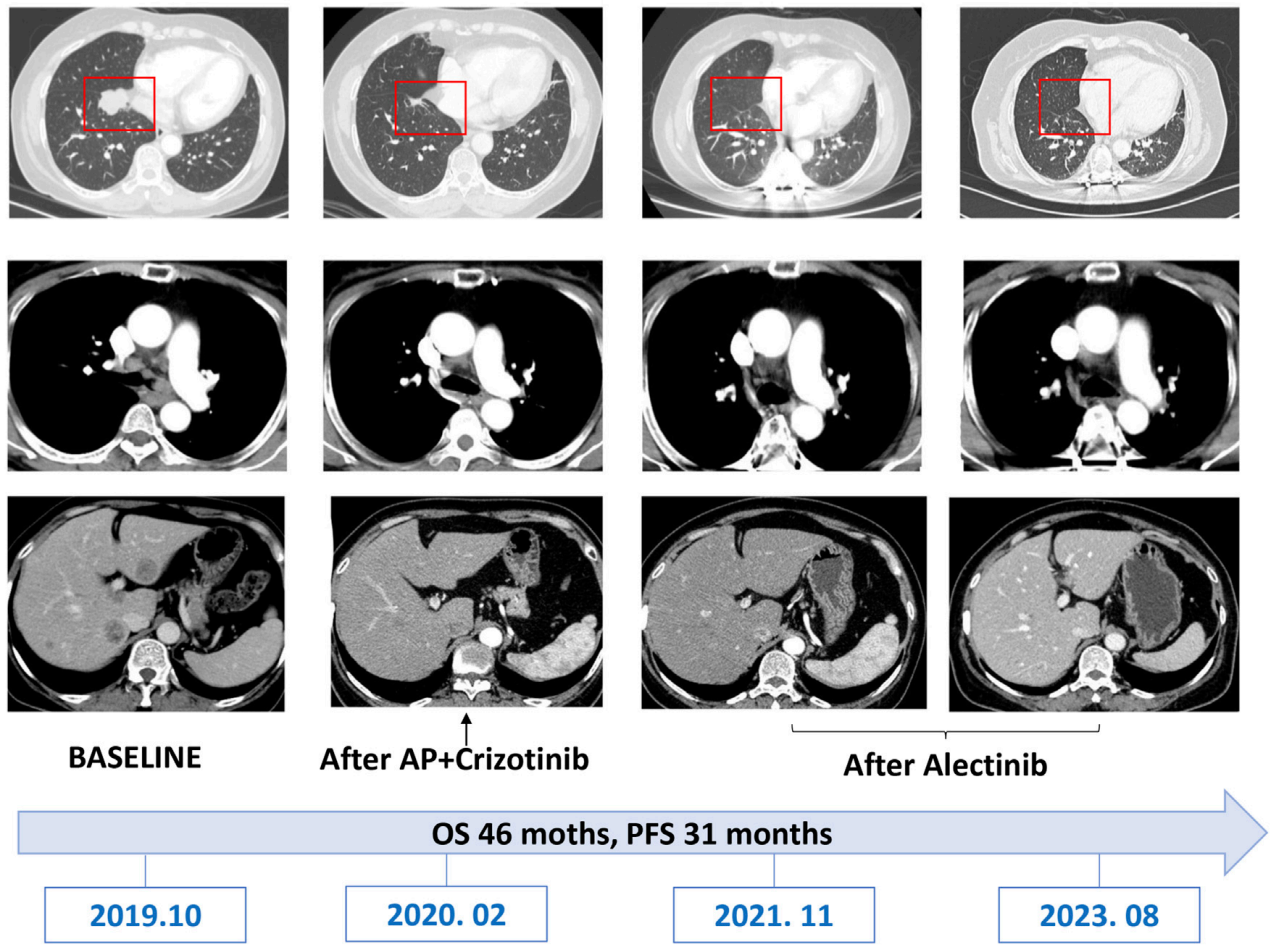
CT Imaging of the reported patients with follow-up.

Two months following the commencement of alectinib treatment, the patient had a PR in all lesions including mediastinal lymph nodes and liver metastases. These promising clinical responses were consistently observed in subsequent follow-up assessments (Figures 1C, 4). Notably, metabolic activity in bone metastases subsided, and the absence of new lesions was confirmed. As of the current stage of manuscript preparation (August 2023), the patient continues to undergo regular follow-up visits, maintaining a state of stable disease during alectinib treatment. Impressively, the patient has achieved a progression-free survival (PFS) exceeding 31 months. Importantly, no significant drug-related adverse events have been observed to date.

## Discussion

To our best understanding, this is the first report documenting a case of stage IV lung adenocarcinoma characterized by a previously unreported *KIF13A-ALK* fusion, identified through NGS, FISH and IHC staining. Furthermore, this study provides the initial clinical substantiation of a patient harboring the *KIF13A-ALK* fusion who benefits from alectinib.

Kinesin family member 13A (*KIF13A*) constitutes a member of the kinesin-3 subfamily of microtubule-based motor proteins, engaging in the vesicular transport from the trans-Golgi network to the plasma membrane. Widely expressed in the central nervous system, *KIF13A* plays an indispensable role in the normal development of intervertebral discs. The fusion protein, *KIF13A-ALK*, retains coiled-coil domains (CCDs) within its N-terminal region, a feature detected in our case (Nakagawa et al., 2000). It is noteworthy that, as observed in other fusion configurations, this *KIF13A* CCD is projected to be integral for the activation of the *ALK* oncogene via homodimerization and kinase activation. Notably, oncogenic fusion between *KIF13A* (exon 18) and the RET gene has been reported in a distinct case report (Zhang et al., 2018); however, the fusion event with *ALK* remains undocumented. It is prudent to regard *KIF13A-ALK* as an oncogenic genetic alteration, meriting inclusion in the catalog of *ALK* fusion variants.

*ALK* fusion events have unequivocally demonstrated their significance as critical targetable oncogenic drivers. The most prevalent *ALK* fusion partner is *EML4-ALK* (88.9%) (Solomon et al., 2014). The expanding adoption of NGS-based testing has augmented the identification of less common *ALK* fusions, resulting in the documentation of over 90 diverse *ALK* fusion protein partners (Ou et al., 2020). However, there persists an ongoing debate surrounding the imperative question of whether infrequent *ALK* fusion variants warrant targeted therapies, given the disparate responses exhibited by diverse *ALK* fusion variants to *ALK* tyrosine kinase inhibitors (TKIs) (Chuang et al., 2021; Sun et al., 2021).

Historically, crizotinib held precedence as the favored first-line treatment for *ALK*-positive NSCLC patients, as it demonstrated superior treatment responses, extended overall survival, and prolonged progression-free survival (Ou et al., 2020). In light of uncertainties regarding the response profile of new variants to *ALK* TKIs, compounded by economic considerations, we opted for chemotherapy as the initial therapeutic strategy. Although the patient displayed a clinical response subsequent to chemotherapy, disease progression manifested after the completion of six cycles. Regrettably, the patient’s condition deteriorated within a month of commencing crizotinib treatment, aligning with previous reports that highlighted the rapid emergence of resistance and consequent tumor relapse as limitations of crizotinib (Chuang and Neal, 2015). Following crizotinib resistance, the patient was transitioned to alectinib therapy. The second-generation inhibitor alectinib exhibited superiority over crizotinib, characterized by lower mean inhibitory concentrations (CI50) against native *ALK* kinase and broader coverage against *ALK* resistance mutations (Samacá-Samacá et al., 2023). Besides, clinicians have previously reported that ALK-positive NSCLS patients achieved long-lasting responses from the use of alectinib (Camidge et al., 2019). Our case demonstrated the potential sensitivity of the new *KIF13A*-*ALK* fusion variant to alectinib, as opposed to crizotinib. Moreover, the patient’s sustained long-lasting response (progression-free survival exceeding 31 months) suggests a rationale for considering alectinib as the optimal therapeutic selection for *KIF13A*-*ALK* fusion-positive cases.

In conclusion, our report stands as the pioneer documentation of the novel *KIF13A*-*ALK* fusion variant, thereby enriching the spectrum of recognized *ALK* fusions. This contribution also bears valuable implications for therapeutic decision-making in this rare subtype. Subsequent work should further elucidate the biological characteristics and optimal treatment interventions for lung cancers bearing the *KIF13A*-*ALK* fusion gene.

## Data Availability

The datasets presented in this study can be found in online repositories. The names of the repository/repositories and accession number(s) can be found in the article/Supplementary Material.

## Abbreviations

*ALK*, Anaplastic lymphoma kinase; CCDs, coiled-coil domains; CT, Computed tomography; FFPE, formalin-fixed paraffin-embedded; FISH, fluorescence *in situ* hybridization; IHC, Immunohistochemical; KIF13A, Kinesin Family Member 13A; NGS, next-generation sequencing; NSCLCs, non-small-cell lung cancers; OS, overall survival; PFS, progression-free survival; PD, disease progression; PR, partial response; TKIs, tyrosine-kinase inhibitors; TTF1, thyroid transcription factor 1.
